# Socio-ecological Determinants of Detectable Viremia among Pregnant People Living with HIV in South Brazil: The Role of Stimulant Use Disorder and Homelessness

**DOI:** 10.1007/s10461-025-04639-5

**Published:** 2025-02-03

**Authors:** Christopher Justin Hernandez, Fernando Echegaray, Kavya Sundar, Lanbo Z. Yang, Mary Catherine Cambou, Eddy R. Segura, Marineide Gonçalves de Melo, Breno Riegel Santos, Ivana Rosângela dos Santos Varella, Karin Nielsen-Saines

**Affiliations:** 1 UCLA David Geffen School of Medicine, Los Angeles, CA, USA; 2 State University of New York, Downstate Health Sciences University, Brooklyn, NY, USA; 3 Department of Obstetrics and Gynecology, Tulane University School of Medicine, New Orleans, LA, USA; 4 Department of Medicine, Division of Infectious Diseases, UCLA David Geffen School of Medicine, Los Angeles, CA, USA; 5 Facultad de Ciencias de la Salud, Universidad Científica del Sur, Lima, Perú; 6 Department of Infectious Diseases, Hospital Nossa Senhora da Conceição, Sistema Único de Saúde, Porto Alegre, Brazil; 7 Department of Epidemiology and Public Health, Hospital Nossa Senhora da Conceição, Sistema Único de Saúde, Porto Alegre, Brazil; 8 Department of Pediatrics, Division of Pediatric Infectious Diseases, UCLA David Geffen School of Medicine, Los Angeles, USA

**Keywords:** Pregnancy, HIV, Viral suppression, Socio-ecological factors, Homelessness, Stimulant use

## Abstract

Pregnant patients living with HIV are a priority group for the recruitment into the HIV healthcare cascade to prevent adverse maternal and neonatal health outcomes. Understanding the structural, interpersonal, and individual factors that are associated with detectable HIV viremia is of importance to guide outreach and intervention priorities. This was a retrospective cohort study of pregnant patients living with HIV who delivered from January 1, 2017, to December 31, 2023, at a tertiary-level hospital and referral institution for HIV care in Porto Alegre, Brazil. The socio-ecological model was used to guide hypothesis testing regarding associations with detectable viremia. In total, 549 patients were included, of whom 110 (20%) were found to have detectable viremia. Significant differences between detectable and undetectable viremia included prenatal care, homelessness, having a sero-different partner, and stimulant use. Multivariable associations included prenatal care (adjusted Risk Ratio [aRR] = 0.20, 95% Confidence Interval [95% CI] = 0.15–0.26), homelessness (aRR = 4.02, 95% CI = 2.74–0.26), stimulant use disorder (aRR = 3.30, 95% CI = 2.23–4.87), crack use (aRR = 2.82, 95% CI = 1.85–4.29), and cocaine use (aRR = 1.89, 95% CI = 1.17–3.06). Intervention research should focus on housing and mental health services, and how to mitigate their impact on HIV healthcare. Intervention research is greatly needed as current tools may not be sufficient to tackle the issue of stimulant use disorder and its effects on ART adherence.

## Introduction

Although there is no cure for HIV, antiretroviral therapy (ART) is highly effective in suppressing viral replication [1]. HIV viremia, defined as the presence of viral particles in blood samples, reflects ongoing viral replication and immune system damage caused by the depletion of CD4 + T lymphocytes [2]. The prompt initiation and consistent use of ART soon after HIV diagnosis preserves immune function and reduces the risk for HIV-related complications for people living with HIV (PLH) [3, 4]. Treatment success is followed longitudinally by measuring an HIV viral load (VL), defined as the number of copies of HIV RNA in a milliliter of blood from PLH [5]. Attaining an undetectable VL occurs when HIV replication is suppressed below levels where viral RNA can be detected and is instrumental in preventing the transmission of HIV infection and halting the progression to Acquired Immune Deficiency Syndrome (AIDS), while enabling immune reconstitution and enhancing health outcomes [4, 5].

HIV is a well-established risk factor for adverse pregnancy and neonatal outcomes, highlighting the importance of identifying and engaging all pregnant people living with HIV (PPLH) in healthcare [6]. Moreover, antiretroviral therapy (ART) reduces the risk of HIV vertical transmission, also known as mother-to-child transmission (MTCT), down to nearly zero, making healthcare engagement essential for the prevention of MTCT [7, 8]. However, even in the absence of vertical transmission, HIV-exposed uninfected (HEU) infants born from PPLH have been shown to have immune dysfunction and increased susceptibility to other infectious diseases with accompanying increases in morbidity and mortality in the first years of life [9]. This phenomenon is particularly prominent in low-and-middle income resource settings [10]. The independent impact of a detectable maternal VL on neonatal immune dysregulation is unclear, however, a mother-HEU infant dyad cohort study demonstrated that infants born from PPLH who did not receive ART before pregnancy had greater alterations to their immune responses compared to infants born from PPLH on ART, indicating the importance of maternal treatment in mitigating the impact of HIV exposure on fetal immune development [11].

Since 1996, the Brazilian Ministry of Health has guaranteed free and universal access to antiretroviral therapy (ART); however, significant challenges remain in addressing the HIV epidemic among PPLH [12]. According to the 2023 Epidemiologic Bulletin of HIV/AIDS from the Brazilian Ministry of Health, the detection rate of PPLH in Brazil increased by 21% between 2012 and 2018, stabilized in 2019, and rose by an additional 3.9% between 2020 and 2022 [13]. These trends vary geographically, with the northeastern (71.7%) and northern (52.8%) regions of the country showing the biggest increases in HIV incidence between 2012 and 2022. Interestingly, even though the southeastern and southern regions of Brazil still maintain the highest overall prevalence of PPLH, there was an overall 13.8% reduction between 2018 and 2022 [10, 13]. In 2022, Porto Alegre, the capital of the Rio Grande do Sul state, had over five times the national average HIV prevalence rate of PPLH (3.1 cases per 1000 live births), estimated at 17.1 cases per 1000 live births. The high HIV prevalence rate identified in the city, combined with the fact that only 67% of PPLH received ART during pregnancy in Porto Alegre suggests that unique barriers to HIV care are at play in this region [14].

It is increasingly recognized that health behaviors are influenced by the individual, social, and structural contexts affecting an individual’s life. These contexts must be taken into consideration when designing interventions to maximize health outcomes [15]. The socio-ecological model (SEM) is a useful framework to identify the multiple-level factors that affect behavioral patterns that relate to ART adherence among PLH [16]. For example, a study among transgender women living with HIV in Los Angeles and San Francisco, California, found that injection drug use, methamphetamine and amphetamine use, homelessness, and sex work were all socio-ecological barriers to achieving an undetectable VL, demonstrating that efforts to promote ART adherence must tackle ongoing syndemic barriers simultaneously [17]. Further, a qualitative study among PPLH in rural Western Kenya found that self-motivation, confidence and resilience, family support, absence or reduced stigma, right provider attitude, and quality of health services were all socio-ecological facilitators for accessing prevention of HIV mother-to-child transmission (PMTCT) services [18]. This study demonstrated that for successful provision of HIV PMTCT services, which are crucial for prevention of HIV vertical transmission, the impact of an individual’s lived experiences on access to care must be taken into consideration, otherwise health care disparities may continue to be perpetuated.

Using a socio-ecological framework to guide our analysis, we examined facilitators and barriers to achieving undetectable VL among PPLH in south Brazil. Specifically, the purpose of this study was to determine the prevalence of detectable viremia among PPLH, characterize vertical transmission events, and identify social and structural factors associated with detectable viremia during pregnancy in a region where HIV prevalence in PPLH is disproportionately high.

## Methods

This is a secondary analysis of a retrospective cohort study using hospital records that are linked to the national epidemiologic surveillance database of PPLH. The study includes all PPLH who delivered from January 1, 2017, to December 31, 2023, at a tertiary-level hospital and HIV referral institution in the city of Porto Alegre, state of RS, south Brazil. Data was extracted from medical records from the government epidemiologic surveillance system, including sociodemographic characteristics. For the purposes of this study, age was stratified as ≤ 20, 21 to 29, or ≥ 30 years. Geographic region was defined as urban, greater metropolitan region, and rural region or outskirts according to publicly available information published by the Porto Alegre City Council. Race or ethnicity was categorized according to hospital records: White, Black, or Parda (mixed race). Although patients could also identify as Asian or Indigenous/Native, none identified as such in this study. We categorized maternal acquisition of HIV infection as one of the following: (1) adult diagnosis through sexual contact, (2) injection drug use (IDU), (3) congenital acquisition, and (4) adult diagnosis but unknown route.

During the study period, all pregnant patients who delivered at this institution in Porto Alegre were tested for HIV using rapid tests during prenatal care and at the time of labor and delivery. Brazilian HIV guidelines recommend that all pregnant patients be tested for HIV during the first trimester of gestation and then again in the third trimester. If patients do not engage in prenatal care, they should be screened for HIV at the time of delivery. The timing of HIV diagnosis in this study was categorized as prior to pregnancy if the patient was aware of their HIV status prior to gestation or during pregnancy if the patient was diagnosed during pregnancy or at delivery. Positive rapid test results were confirmed using either the HIV antibody enzyme-linked immunosorbent assays (ELISAs) or the immunoblot for HIV-1 antigens (p24, gp41, gp120, and gp160) and HIV-2 antigen gp36 as recommended by Brazilian HIV guidelines. For this study, we excluded patients who delivered at home. HIV-specific parameters were extracted from medical records, including the date of VL collection. The maternal VL value obtained closest to the date of delivery was used for this analysis. VL dates ranged from ten months prior to delivery to two months postpartum. PPLH with a consistently undetectable VL may have had only one VL assay performed during pregnancy and were assumed to have an undetectable VL at the time of delivery by their providers, thus no VL at delivery might have been available for some patients. The two-month window period postpartum allowed PPLH who only had VL measures performed at or immediately post-delivery because of late presentation to care to be included in this study. We used a national laboratory database known as Sistema de Controle de Exames Laboratoriais (SISCEL), which contains HIV VL information for all persons living with HIV in Brazil, to cross-reference our VL data. The use of this national surveillance system allowed us to access VL information for patients who delivered at our institution but received HIV care elsewhere. There were two instances in which maternal VL could not be obtained through either hospital records or SISCEL because they declined VL testing during hospitalization. Because neither patient was receiving ART and one vertical transmission case occurred to one of these patients, we included both patients in the group with detectable VL.

The primary outcomes were stratified according to the following definition of viral suppression; undetectable VL, defined as < 200 copies/mL and (2) detectable VL defined as ≥ 200 copies/mL. The secondary outcome was HIV vertical transmission; however, we focused on descriptive statistics since there were only two documented cases. National perinatal guidelines were used to identify infants with HIV infection. Confirmed perinatal HIV infection was present if any of two VL values measured during the first year of life were detectable using an RNA PCR method. If infants were lost to follow up (LTFU) for the first 15 months of age, after which maternal anti-HIV antibodies in the child wane, a positive HIV antibody ELISA screen followed by confirmatory VL testing was diagnostic of vertical transmission. Infants were considered HIV negative if they had undetectable VL beyond ≥ 3 months of life and/or a negative HIV antibody ELISA test at 15 months of age. Infants without postnatal confirmation of their HIV status were considered lost to follow-up.

**Fig. 1:**
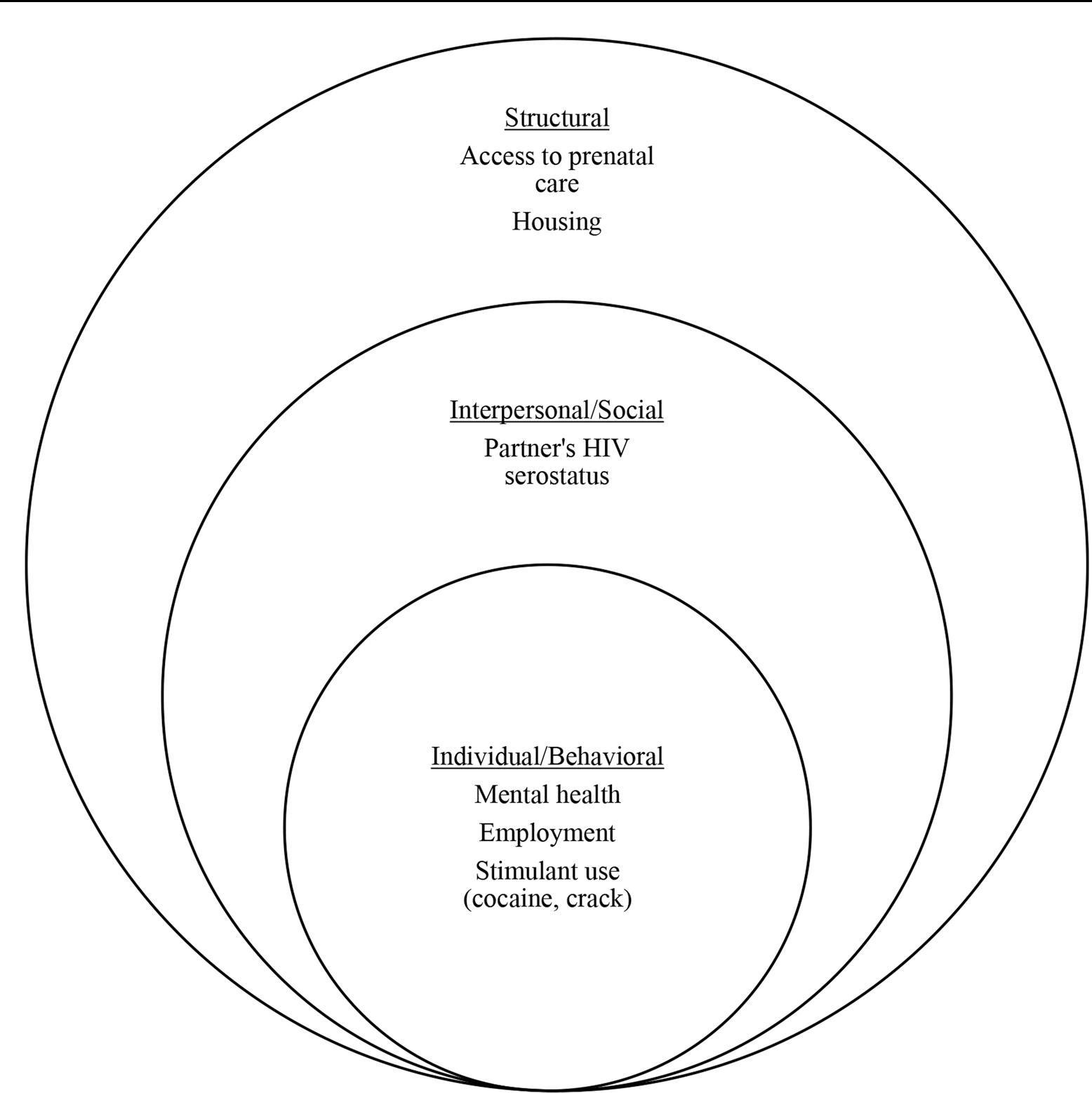
Hypothesized socio-ecological associations with detectable viremia among pregnant people living with HIV. Note, the associated factors do not cover the spectrum of factors associated with structural, social, and individual determinants of health

To examine the hypothesized SEM factors (Fig. 1), we obtained information related to mental health, stimulant use, housing status, and ART adherence from patients’ medical records. With the understanding that there is a bidirectional association between the different levels and that factors can be categorized differently depending on the analytical lens being employed, we loosely categorized access to prenatal care and homelessness during pregnancy as structural factors. Homelessness during pregnancy was identified by one of the following means: (1) if clinical notes by the street clinic/ “Consultório na Rua” were present in medical records, (2) if patient’s self-reported being unhoused any point during pregnancy, or (3) if social worker notes identified unstable housing during pregnancy, which included living in temporary shelters or moving frequently between friend’s or family’s houses.

We hypothesized that a partner’s serostatus could affect the household support for HIV care, therefore we examined the association, if any, between partner’s HIV serostatus and ART adherence in PPLH. From a public health standpoint, a partner’s serostatus is important in assessing the risk of HIV sexual transmission in case the index partner has suboptimal adherence to ART [19]. Partner categories included sero-same, sero-different, partners who refused or delayed HIV testing, absence of a stable partner, defined as a partnership lasting less than 3 months or absence of any partner.

The individual factors included in the study are: (1) diagnosed mental health condition prior to date of delivery, including depression, stimulant use disorder, bipolar disorder, cognitive impairment, and any cluster personality disorders, and (2) reported stimulant use (crack and/or cocaine). Mental health information was retrieved from (1) psychiatry medical record notes written prior to or during prenatal care or at the time of delivery or (2) by the problem list written by obstetrics or infectious disease medical notes. Stimulant use disorder was assessed by a psychiatrist. Stimulant use was assessed from ID/obstetrics notes: never user, active user during pregnancy, abstinence for at least 12 months prior to date of delivery, or abstinence for more than 12 months prior to delivery. Because there was no standardized way of reporting stimulant use among physicians, we relied on a diagnosis of stimulant use disorder to more robustly identify problematic use impacting daily life. Patients with no medical records beyond procedure notes (no past medical or social history) were classified as unknown and excluded from statistical analysis.

### Statistical Analysis

Descriptive statistics were used to summarize demographic characteristics and clinical information. Bivariate two-tailed chi-square tests, when *n* ≥ 5, and Fisher’s exact tests, when *n* < 5, were used to determine whether there were statistically significant differences in sociodemographic factors and detectable VL at the *p* ≤ 0.05 level.

Given that several PPLH had multiple pregnancies during the time of the study, we used the generalized estimating equations (GEE) because of the lack of independence (correlations between observations within a subject [20]. Modified Poisson regression was used to estimate the risk ratio of detectable HIV viral load by demographic and risk subgroups [21]. The association of individual SEM factors with a detectable VL was investigated by building separate GEE modified Poisson regression models for each individual SEM exposure of interest while controlling for age, educational level, timing of the HIV diagnosis, and maternal HIV infection acquisition route, each of which were associated with a detectable VL at the bivariate level [22]. We decided to include only confounders that were significant at the bivariate level to prevent over-adjustment of the results. Delivery by C-section, which was significant at the bivariate level, was not controlled as the medical decision was made at the time of delivery and decided by the maternal viral load results [23]. In addition, because our analysis was restricted to VL and not maternal or neonatal outcomes, mode of delivery was not included in our analysis. Due to small sample sizes that precluded granular analyses, we collapsed stimulant use to never user or lifetime use (any use coded as 1). Models were checked for collinearity using Variance Inflation Factor and model fitness was verified by the Pseudo R-squared result. Corresponding subjects exhibiting missingness of certain datapoints for certain variables were, by standard default settings of any statistical software, not included in each corresponding analyses. All analyses were conducted using STATA version 14 (College Station, TX).

## Results

Among the total of 23,486 pregnant patients seen at this site during the study period, 549 (2.3%) PPLH were identified and included in this study. Slightly less than half (49%) of PPLH were over 30 years of age, 42% were between 21 and 29 years of age and 10% were 20 years of age or under (Table 1). The majority of PPLH (53%) identified as white, a third (31%) identified as Black, and 16% identified as multiracial. Of note, the demographics of Porto Alegre are approximately 90% white. Eighty percent of PPLH had less than high school completed, and 20% completed a high school or higher degree. Most PPLH (74%) resided in the urban region of Porto Alegre, 21% in the greater metropolitan region, and 5% in rural areas.

The yearly totals for pregnant patients seen at this site are as follows: 3,256 in 2017, 3,649 in 2018, 3,780 in 2019, 3,564 in 2020, 2,901 in 2021, 3,192 in 2022, and 3,144 in 2023. HIV prevalence showed a slight overall decrease during the study period, peaking at 2.8% in 2017, followed by 2.5% in 2018, 2.3% in 2019, 2.2% in 2020, rising slightly to 2.5% in 2021, then dropping to 2.0% in 2022, and reaching a low of 1.9% in 2023. Among all PPLH, 20% (95% CI: 16.7–23.4%) were found to have a detectable VL at or near the time of delivery. The yearly prevalence of detectable VL (95% CI) also demonstrated a nonsignificant downward trend: 27.2% (17.9–36.4%) in 2017, 18.7% (10.5–26.8%) in 2018, 18.2% (10.0–26.4%) in 2019, 25.3% (15.5–35.1%) in 2020, 15.1% (6.7–23.5%) in 2021, 16.9% (7.6–26.3%) in 2022, and 16.4% (6.5–26.0%) in 2023.

The median (interquartile range [IQR]) VL values among those with a detectable VL were as follows: 6,775 copies/mL (1,002–22,342) in 2017, 2,437 copies/mL (693–22,970) in 2018, 2,493 copies/mL (800–5,520) in 2019, 4,930 copies/mL (2,189–39,320) in 2020, 1,500 copies/mL (360–21,227) in 2021, 9,584 copies/mL (1,502–58,285) in 2022, and 3,678 copies/mL (2,662–16,297) in 2023. The overall median (IQR) VL throughout the study period was 3,569 copies/mL (1,002–21,683).

As shown in Table 1, most PPLH acquired HIV infection during adulthood, likely through sexual contact, while less than 1% acquired through injection drug use. Seven percent of PPLH were confirmed to have acquired infection perinatally, while another 6% of PPLH were diagnosed with HIV in adulthood, although the means of transmission were unclear, and perinatal infection could not be ruled out. Most PPLH (83%) were diagnosed with HIV infection prior to pregnancy, while the remaining 17% were diagnosed in pregnancy. Overall, one half (50%) of PPLH, *n* = 135 delivered via C-section; however, among those with detectable viremia, 67% delivered by C-section.

Ninety-five percent of PPLH received prenatal care during pregnancy, as shown in Table 1. Five percent of patients reported homelessness. Forty-five percent reported a sero-different partner, 32% a sero-same partner, 3% had a partner who refused or delayed an HIV test, and 16% had no stable partner. Approximately 39% of PPLH were employed. The most common mental health condition recorded was depression (18%), followed by stimulant use disorder (10%), bipolar disorder (5%), cognitive impairment (2%), and any other mental health condition (3%). Crack use was reported by 10% and cocaine by 9% of participants (Table 1).

In terms of neonatal outcomes, there were 533 (97.1%) live births and 16 (2.9%) documented fetal or infant deaths that precluded determination of HIV status. Among live births, 88.6% of infants were retained in pediatric healthcare while 11.4% of infants were lost to follow up (LTFU) without determination of HIV status. Importantly, 36.1% of infants with LTFU were born from PPLH with a detectable VL compared to only 17.8% of infants who were retained in care being born from PPLH with a detectable VL. Among infants retained in care, 2 (0.4%) were found to have HIV infection, one occurring in 2021 and the other in 2023. Among the two PPLH who had MTCT events, one had a detectable VL, and the other did not have a VL performed at any time before or during pregnancy or after delivery. Only one of the mothers who transmitted HIV received prenatal care.

As shown in Table 1, detectable VL was more frequently identified in patients 21–29 years and patients 30 + years (*p* = 0.009). Also, detectable VL was more prevalent in patients with less education (*p* = 0.015), who acquired HIV perinatally (*p* = 0.003), and those diagnosed with HIV during pregnancy (*p* = 0.024) At the structural level, detectable VL was associated with lack of prenatal care (*p* = 0.001) and homelessness (*p* = 0.001). At the interpersonal level, associations with detectable viremia and a partner of sero-different status were identified (*p* = 0.006). Finally, at the individual level, detectable VL was associated with stimulant use disorder (*p* = 0.001), and a lifetime use of crack or cocaine (*p* = 0.001).

As shown in Table 2, independent predictors considered risk factors for a detectable VL included prenatal care, as a protective factor, (adjusted Risk Ratio [aRR] = 0.20, 95% Confidence Interval [95% CI] = 0.15–0.26). Conversely, the present study identified the following risk factors for a detectable VL: experiencing homelessness (aRR = 4.02, 95% CI = 2.74 – 0.26), stimulant use disorder (aRR 162 = 3.30, 95% CI = 2.23–4.87), and lifetime use of crack (aRR = 2.82, 95% CI = 1.85–4.29) and cocaine (aRR = 1.89, 95% CI = 1.17–3.06).

## Discussion

This study revealed multiple socio-ecological associations with maternal detectable HIV viremia, at or near delivery in south Brazil, the region burdened with the highest prevalence of HIV infection among PPLH. We found that prenatal care was a protective factor for PPLH, reducing the risk of a detectable VL by 81%, while experiencing homelessness or unstable housing increased the risk by nearly 300%. Living with a stimulant use disorder, specifically the use of crack or power cocaine, significantly increased the risk of detectable VL during pregnancy. Although only two cases of vertical transmission occurred among PPLH in this study, it is important to note that more than a tenth of neonates born to PPLH, more than a third of whom had detectable viremia, were LTFU and therefore HIV MTCT could not be assessed. This is concerning given that the risk for HIV infection is potentially much higher for these infants and may be discovered only once AIDS-defining illnesses or other HIV-associated disorders manifest. Finally, this study found a promising downward trend of PPLH, likely a reflection of both lowered incidence among women of child-bearing age and a population of PLH that is aging out of their reproductive years. There was also a downward trend of PPLH with a detectable VL over the study period, an encouraging sign of increased ART uptake among PPLH in this region. However, major gaps remain that must be addressed if eliminating MCTC and obtaining better HIV-related health outcomes in south Brazil are to be achieved.

In our study, the majority of PPLH received prenatal care and those who did were more likely to have an undetectable VL. This likely relates to the overlap in barriers to accessing prenatal care and HIV care, a point further elucidated by the lower rate of prenatal care among those with a detectable VL. In Brazil, prenatal care has been integrated into maternal health to eliminate vertical transmission [24]. Our study site is a pioneer in the introduction of the multidisciplinary prenatal care model for PPLH, named *Unidade de Prevenção da Transmissão Vertical*, which offers services that include infectious disease management, psychological and psychiatric care, and aesthetics as they relate to the side effects of ART (e.g. lipodystrophy from the use of protease inhibitors) [25, 26]. This tailored care, which may encourage physicians and mothers to focus beyond fetal health and integrate maternal needs, has been shown to optimize care, highlighted by the reduction of vertical transmission of HIV to less than 1% at this institution [25]. Further, the provision of mental healthcare promotes wellbeing, ensuring that there is ongoing support and motivation in the face of variable emotional stability for many PPLH [27].

There are myriad reasons why PPLH may not be accessing prenatal care in Brazil, ranging from, but not limited to, lack of transportation, time, or other internal motivations [28]. As a tertiary public healthcare center, our study site serves a population that is of generally lower income, so even with free healthcare, factors outside of the institution are likely significant ongoing barriers. In fact, while reviewing medical records, we noted that some of the reasons PPLH stated for not accessing prenatal care included having to work during clinic hours, inadequate transportation, and fear of leaving their homes due to neighborhood violence. Qualitative studies among PPLH who had no or poor history of accessing prenatal care may reveal solutions to increase uptake, whether it is shifting clinic hours to accommodate working people or providing direct transportation in and out of neighborhoods. These solutions should be patient-centered and focused on tackling the enduring barriers to visiting prenatal care clinics.

Although a minority of PPLH experienced homelessness, we found that it represents a significant risk factor for a detectable VL. Homelessness has been shown to deepen disparities in HIV incidence and related HIV-related outcomes [29, 30]. From a practical standpoint, homelessness presents challenges to privacy and limits options for adequate storage of medications, potentially preventing individuals from maintaining ART on hand [31]. In south Brazil, a street clinic called “Consultório na Rua”, aims to reach unhoused people to provide comprehensive health services, including ART among those living with HIV [32]. Despite these efforts, a qualitative study among homeless pregnant people in Porto Alegre, Brazil highlighted that discrimination and stigma were highly prevalent when accessing healthcare, even at the time of labor and especially after delivery [33]. Patients’ negative experiences may fuel skepticism towards the healthcare system, preventing care and follow-up for both infants and mothers.

To tackle the effects of homelessness, there needs to be concerted efforts to provide housing and address the ongoing prejudice that the healthcare system imposes on patients. Housing advocates have recommended that accessible housing be provided without coercive requirements (e.g. random urine toxicology screens, curfews, prohibiting visitors), which act as barriers to housing for individuals may not find it compatible with daily life [34]. Empathy, much like any skill in healthcare, can be taught and used to address the stigma that creates an unsafe environment for patients experiencing homelessness [35]. One commonly used definition of empathy in healthcare research is that of Mercer and Reynolds, which they defined as the ability to understand a patient’s situation, perspective, and feelings and then be able to communicate that understanding in a helpful and therapeutic way [36]. In a midwestern region of the United States, the deployment of a novel inter-professional curriculum for healthcare trainees designed to address the needs of homeless patients demonstrated that trainees developed greater empathy for their patients and provided the trainees with tools to more rigorously address social determinants of health [37]. Similar pilot programs or related courses for healthcare providers in this region may help reach PPLH who are experiencing homelessness and enhance their experiences with healthcare systems to promote engagement with HIV care.

One-fifth of PPLH reported use of crack or cocaine prior to delivery, and one-tenth were diagnosed with a stimulant use disorder by a psychiatrist or other healthcare provider and were significantly associated with an increased risk of having a detectable VL. The high prevalence of crack and cocaine use in this study is expected as population-wide surveys have demonstrated Brazil to be one of the nations with greatest annual consumption rates of stimulants [38]. Stimulant use impacts ART adherence in multiple ways. It affects cognitive health and the ability to maintain the appropriate medication routines and modifies the perceived risks of HIV infection and the importance of treatment adherence [39, 40]. The city of Porto Alegre has eight *Centros de Atenção Psicossocial Álcool e Drogas (Centers dedicated to providing psychosocial support to users of alcohol and drugs)* (CAPS AD) which help treat chemical dependency, including alcohol, tobacco, crack, cocaine, and marijuana use [41]. They are open to anyone who requests their services and patients are triaged into different lengths of treatment, but all are guided through the gradual reduction of chemical dependency to the resumption of daily life.

Unfortunately, pharmacotherapy for stimulant use is mostly restricted to managing withdrawal symptoms and no medications have been shown to provide a benefit for long-term remission [42]. Similarly, a Cochrane review for psychosocial interventions for stimulant use disorder found that these interventions may help prevent patients from dropping out of programs prematurely and help prolong periods of abstinence when compared to no treatment, but do not demonstrate benefit for long-term abstinence [43]. Contingency management, a strategy that rewards desired behaviors or outcomes (e.g. cash reward for attending counseling sessions), is one of the few evidence-based interventions shown to reduce stimulant use and may be a potential strategy to address stimulant use disorder in PPLH [44]. A 2023 systematic review of the efficacy of contingency management for improving adherence to ART found a significant increase in adherence while patients were in the intervention phase; however, this benefit was lost once the incentives were ceased [45]. If permanent funding of contingency management programs is not a realistic goal, then interventions may be employed to promote and prolong periods of abstinence that coincide with the peripartum period, when the risk for vertical transmission is highest. These findings add to the importance of identifying novel ways of addressing stimulant use disorders among PLH as our current arsenal may not be enough to tackle this co-occurring epidemic.

Adherence to ART among patients with stimulant use disorder cannot be entirely assigned to the individual, however, as stigma and discrimination by healthcare institutions and providers present important barriers to healthcare [46]. Physicians and other healthcare staff with stigmatizing practices may deprioritize the needs of PPLH who have a concomitant stimulant use disorder, thereby predisposing them to poor experiences in healthcare. Such events, much like those experienced by people experiencing homelessness, may discourage them from seeking regular healthcare. There are ongoing studies that show effectiveness in harm reduction strategies that emphasize approaches to strengthen provider-patient relationships, avenues that can enhance patient-centered approaches and outcomes in HIV care [47].

Our study has limitations. One limitation is the assumption that detectable viremia is a consequence of not maintaining adequate ART; however, other reasons like treatment failure due to viral resistance may be causative. Nonetheless, the review of records indicated that viral susceptibility profiles with viral genotypes were examined, and the ART regimen modified if there were concerns for viral resistance. Thus, the predominant reason for detectable VL was the failure of starting or adhering to ART. Another limitation was the retrospective nature of the study where causality between associations cannot be fully ascertained. For example, it may be that HIV infection and the societal stigma it carries predisposes PPLH to the risk of homelessness if they are disowned by family and/or engage in substance use to cope with the diagnosis. In many situations, however, substance abuse and mental health disorders tend to predate HIV acquisition. Nevertheless, despite the comprehensive collection of behavioral data by obstetricians, infectious disease physicians, psychiatrists, and social health workers, there may be reporting bias by patients who do not want to disclose social history, such as stimulant use or housing status. This bias may lead to an underestimation of the true prevalence of these factors, which may affect the level of the association with a detectable VL. Importantly, this study aimed at analyzing VL at or near delivery, which could present limitations on categorization. We assume that PPLH with an undetectable VL early during pregnancy and no follow-up VL at a time nearer to delivery had an undetectable VL during delivery. Although concerns about ART adherence would have been documented on patient medical records, we could still have missed PPLH who ceased ART adherence after an undetectable VL. Also, PPLH who had a diagnosis of HIV late in pregnancy may have been fully motivated to start and remain on ART but did not have enough time to achieve an undetectable VL, thereby being categorized with the detectable group. Another important limitation is the relatively small subgroups for SEM factors of interest, notably homelessness. This may affect the robustness of these findings and obscure the true independent impact of housing status on healthcare. Despite these limitations, this study demonstrates important ongoing socio-ecological associations with detectable HIV VL among PPLH and provides further evidence for the need to strengthen public health interventions. Despite increased efforts in Brazilian policy to address the HIV epidemic, challenges remain in reaching these populations in south Brazil. Closing the gaps in this battle will require understanding the barriers and facilitators to achieving optimal HIV care.

## Conclusion

In south Brazil, stimulant use disorder, homelessness, and lack of prenatal care are persistent barriers to achieving an undetectable VL among PPLH. Intervention research focusing on ways to better engage PPLH with concomitant stimulant use disorder in HIV care needs to be explored as current tools may not suffice to address this problem. Furthermore, policy changes that center on the welfare of PPLH experiencing homelessness, especially in the context of the lived realities in south Brazil, can provide the needed stability among this at-risk population to remain in healthcare. Finally, prenatal care is vital to preventing HIV MTCT and optimizing maternal health, and understanding why accessibility can be difficult for some populations can inform ways to increase access.

## Figures and Tables

**Table 1:** Demographic and clinical information of all pregnant people living with HIV (PPLH) (center left) and PPLH with detectable viral load (VL) at the time of delivery (center right) who delivered at the study institution in south Brazil from 2017–2023 (*n* = 549). The right-most column represents statistical differences of demographic and clinical information between PPLH with and without a detectable VL

	Total *n* = 549 (%)	Detectable VL *n* = 110 (%)	*p*-value^a^

Age at time of hospitalization (*n* = 549)			
≤ 20	53 (9.7)	16 (14.6)	0.009
21–29	229 (41.7)	54 (49.1)	
≥ 30	267 (48.6)	40 (36.4)	
Region (*n* = 549)			
Urban	408 (74.3)	89 (80.9)	0.116
Greater metropolitan region	114 (20.8)	19 (17.3)	
Rural region/outskirts	27 (4.9)	2 (1.8)	
Race (*n* = 548)			
Black	170 (31.0)	27 (24.8)	0.094
Multiracial	88 (16.1)	24 (22.0)	
White	290 (52.9)	58 (53.2)	
Education level (*n* = 547)			
Less than high school	436 (79.7)	96 (88.1)	0.015
High school completion	111 (20.3)	13 (11.9)	
HIV Viral Load (copies/ml)			
Median (IQR)	0 (0, 51)	3,691 (1,002, 22,342)	
Maternal HIV acquisition route (*n* = 549)			
Adult diagnosis (likely sexual)	474 (86.3)	86 (78.2)	0.003
Injection drug use (IDU)	4 (0.73)	0 (0.0)	
Congenital acquisition	38 (6.9)	16 (14.6)	
Adult diagnosis, unknown route	33 (6.0)	8 (7.3)	
Timing of HIV diagnosis (*n* = 541)			
Prior to pregnancy	451 (83.4)	83 (76.2)	0.024
During pregnancy	90 (16.6)	26 (23.9)	
Delivery type (*n* = 546)			
Vaginal delivery	276 (50.6)	36 (33.0)	0.001
C-section	270 (49.5)	73 (67.0)	
Vertical transmission documented			
Yes (among those not LTFU *n* = 472)	2 (0.4)	2 (2.4)	***
*Structural*			
Access to prenatal care (*n* = 548)			
No	27 (4.9)	22 (20.2)	0.001
Yes	521 (95.1)	87 (79.8)	
Housing status (*n* = 528)			
Stable housing	504 (95.5)	85 (84.2)	0.001
Unstable/ Experiencing Homelessness	24 (4.6)	16 (15.8)	
*Interpersonal*			
Partner serostatus (*n* = 548)			
Serosame	173 (31.6)	33 (30.0)	0.006
Serodiffernt	247 (45.1)	38 (34.6)	
No fixed partner	89 (16.2)	25 (22.7)	
Partner declined HIV test	15 (2.7)	4 (3.6)	
Has partner, no information	24 (4.4)	10 (9.1)	
*Individual*			
Employment (*n* = 539)			
Unemployed	331 (61.4)	72 (67.3)	0.163
Employed	208 (38.6)	35 (32.7)	
Mental Health (*n* = 528)			
Depression	97 (18.4)	19 (19.2)	0.794
Stimulant Use Disorder	50 (9.5)	24 (22.0)	0.001
Bipolar Disorder	27 (5.1)	4(3.7)	0.425 ^b^
Cognitive impairment	8(1.5)	1 (1.0)	
Other mental health condition	18 (3.4)	3 (3.0)	
Stimulant (*n* = 543)			
Crack			
Current use	27 (5.0)	14 (13.0)	0.001
Cessation in the past 12 months	3 (0.6)	1(1.0)	
Cessation 12 + months	22 (4.0)	6 (5.6)	
Cocaine			
Current use	24 (4.4)	9 (8.3)	0.001
Cessation in the past 12 months	5 (1.0)	2 (1.9)	
Cessation 12 + months	19 (3.5)	6 (5.6)	

Fisher’s exact tests were conducted if any one cell ≤ 5, otherwise Chi square tests were used

Bipolar personality disorder, cognitive impairment, and other mental health conditions collapsed for analysis

The sixteen infant mortalities prior to follow-up completion period were dropped from analysis

**Table 2:** Unadjusted and adjusted multivariable regression analyses of socio-ecological risk factors and a detectable maternal HIV VL during pregnancy at delivery

Variable	Unadjusted Risk Ratio (95% CI),	Adjusted Risk Ratio (95% CI), Models 1–7)

*Structural*		
Access to prenatal care		
No	Reference	Reference
Yes	0.21 (0.16–0.27)	0.20 (0.15–0.26)
Housing status		
Stable housing	Reference	Reference
Unstable/Homeless	3.86 (2.64–5.64)	4.02 (2.74–5.91)
*Interpersonal*		
Partner serostatus		
Seroconcordant	Reference	Reference
Serodisocordant	0.81 (0.53–1.24)	0.75 (0.49–1.15)
No fixed partner	1.47 (0.94–2.29)	1.41 (0.92–2.18)
Partner declined HIV test	1.40 (0.57–3.42)	1.23 (0.47–3.22)
*Individual*		
Employment		
Unemployed	Reference	Reference
Employed	0.78 (0.54–1.12)	0.85 (0.59–1.22)
Mental Health ^a^		
Depression	1.07 (0.68–1.68)	1.19 (0.76–1.87)
Stimulant Use Disorder	3.06 (2.11–4.42)	3.30 (2.23–4.87)
Other	0.79 (0.43–1.46)	0.83 (0.45–1.53)
Stimulant Use		
Crack	2.46 (1.64–3.69)	2.82 (1.85–4.29)
Cocaine	1.94 (1.23–3.06)	1.89 (1.17–3.06)

All models were adjusted by age, educational level, timing of HIV diagnosis, and maternal HIV infection acquisition route

Boldface indicates statistical significant at the *p* ≤ 0.05 level
